# Bavachin from *Psoralea corylifolia* Improves Insulin-Dependent Glucose Uptake through Insulin Signaling and AMPK Activation in 3T3-L1 Adipocytes

**DOI:** 10.3390/ijms17040527

**Published:** 2016-04-08

**Authors:** Hyejin Lee, Hua Li, Minsoo Noh, Jae-Ha Ryu

**Affiliations:** 1 College of Pharmacy and Research Center for Cell Fate Control, Sookmyung Women’s University, 52 Hyochangwongil, Yongsan-gu, Seoul 140-742, Korea; u9698115@naver.com (H.L); cooldog227@hotmail.com (H.L.); 2 Natural Products Research Institute, College of Pharmacy, Seoul National University, 1 Gwanak-ro, Gwanak-Gu, Seoul 151-742, Korea; minsoonoh@snu.ac.kr

**Keywords:** diabetes mellitus, glucose uptake, *Psoralea corylifolia*, 3T3-L1, adipogenesis

## Abstract

The fruit of *Psoralea corylifolia* L. (Fabaceae) (PC), known as “*Bo-Gol-Zhee*” in Korea has been used as traditional medicine. Ethanol and aqueous extracts of PC have an anti-hyperglycemic effect by increasing plasma insulin levels and decreasing blood glucose and total plasma cholesterol levels in type 2 diabetic rats. In this study, we purified six compounds from PC and investigated their anti-diabetic effect. Among the purified compounds, bavachin most potently accumulated lipids during adipocyte differentiation. Intracellular lipid accumulation was measured by Oil Red-O (ORO) cell staining to investigate the effect of compounds on adipogenesis. Consistently, bavachin activated gene expression of adipogenic transcriptional factors, proliferator-activated receptorγ (PPARγ) and CCAAT/enhancer binding protein-α (C/EBPα). Bavachin also increased adiponectin expression and secretion in adipocytes. Moreover, bavachin increased insulin-induced glucose uptake by differentiated adipocytes and myoblasts. In differentiated adipocytes, we found that bavachin enhanced glucose uptake via glucose transporter 4 (GLUT4) translocation by activating the Akt and 5′AMP-activated protein kinase (AMPK) pathway in the presence or absence of insulin. These results suggest that bavachin from *Psoralea corylifolia* might have therapeutic potential for type 2 diabetes by activating insulin signaling pathways.

## 1. Introduction

Type 2 diabetes mellitus (DM) arises from a defect in insulin usage by metabolic organs, such as muscle, liver, and adipose tissue. High blood glucose level is a clinical marker of DM together with gain of body and organ weights and insulin resistance. Decreased glucose transport is related to a risk for DM, thus, it is important to control blood glucose level to improve insulin sensitivity.

Mouse 3T3-L1 pre-adipocytes are a well-established cell line to examine insulin sensitizing activity of anti-diabetic compounds [1]. Hormonal treatments stimulate differentiation of pre-adipocytes into mature adipocytes, concomitant with lipid accumulation. As a marker of adipocyte differentiation, increased intracellular lipid accumulation suggests improved insulin sensitivity [2].

*Psoralea corylifolia* L. (Fabaceae) (PC) is a widely used multi-functional medicinal herb in Asian countries. The major constituents of PC, such as bakuchiol, psoralen, bavachin, corylifolin, coumarins, daidzin, and corylin, show antioxidative, anti-tumor, anti-bacterial, and protective effects on cutaneous complaints, impotence, and hepatic injury [3]. Several studies have examined the anti-diabetic efficacy of PC in a type 2 DM animal model. The ethanol extract of PC exhibit anti-hyperglycemic and antioxidative effects in type 2 DM rats by increasing plasma insulin level and decreasing blood glucose, glycosylated hemoglobin, and total plasma cholesterol levels [4]. Water extract of PC protects β cells against oxidative stress [5] and improves insulin sensitivity in streptozocin-induced diabetic rats [6]. Another study revealed that methanol extract of PC increased glucose uptake by yeast cells [7]. Consequently, a PC extract could be a potential candidate for treating type 2 DM by enhancing insulin-induced glucose uptake.

Bakuchiol from *Otholobium pubescens* (fabaceae), one of main compound of PC, was reported to have anti-hyperglycemic activity in type 2 DM rats [8], but no anti-diabetic compound from PC has not been studied. In the present study, we tried to find anti-diabetic compounds from PC and disclose its possible molecular mechanisms.

## 2. Results

### 2.1. Purification of Peroxisome Proliferator-Activated Receptorγ (PPARγ) Ligands from Psoralea corylifolia

**Figure 1:**
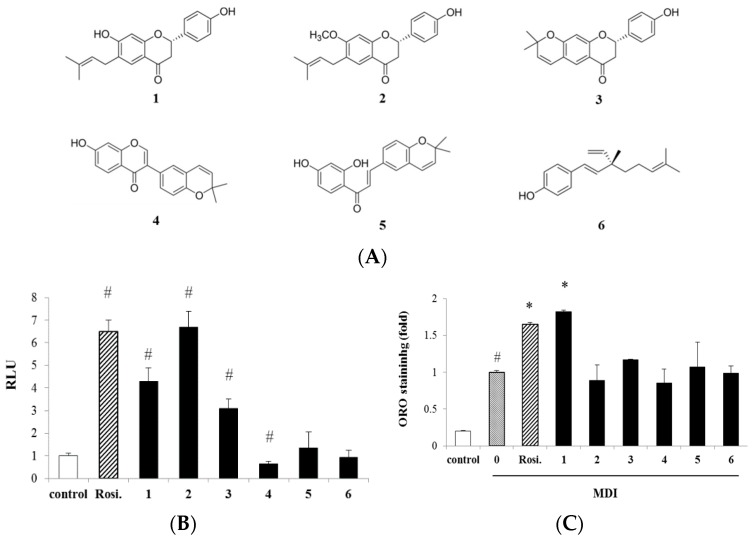
Effect of compounds on transactivation of peroxisome proliferator-activated receptorγ (PPARγ) and adipocytes differentiation: (**A**) Chemical structures of compounds **1**–**6** isolated from *Psolarea corylifolia* (PC); (**B**) Effect of compounds on transactivation of PPARγ. CV-1 cells were transiently transfected with plasmid mixture containing PPARγ expression vector and thymidine kinase-PPAR response element-luciferase (tk-PPRE-Luc) vector, and then treated with compounds (10 µM). Data are presented as relative luciferase activity (RLU) divided by the β-galactosidase activity; (**C**) Effect of compounds on adipocyte differentiation. 3T3-L1 cells were differentiated in the presence of compounds **1**–**6** (2 µM) or rosiglitazone (1 µM). At differentiation Day 8 (D8), cells were stained with Oil Red O (ORO), and lipid accumulation was quantified by measuring absorbance. Data are expressed as mean ± standard deviation (SD). Control, media control; 0, MDI (the mixture of isobutyl-methylxanthine, dexamethasone, and insulin); Rosi., rosiglitazone; **1**–**6**, compounds **1**–**6**. ^#^ *p* < 0.001 *vs.* control; * *p* < 0.01 *vs.* MDI.

Six compounds were purified from the ethyl acetate soluble fraction of PC as peroxisome proliferator-activated receptorγ (PPARγ) ligands, and their structures were identified as bavachin (**1**), bavachinin (**2**), 7,8-dihydro-8-(4-hydroxyphenyl)-2,2-dimethyl-2*H*,6*H*-benzo-[1,2-b:5,4-b′] dipyran-6-one (**3**), corylin (**4**), kanzonol B (**5**), and bakuchiol (**6**) by spectroscopic analysis [9,10] (Figure 1A).

To discover insulin sensitizer, we purified six compounds from ethyl acetate fraction of PC and measured their PPARγ transcriptional activity in CV-1 cells (Figure 1B). Among the tested compounds, compounds **1**, **2** and **3** increased PPARγ transcriptional activity at 10 µM, while compound **4** significantly decreased when compared with control group. Compounds **5** and **6** showed no significant activity.

Next, the adipogenic activity of six compounds was evaluated using Oil Red O (ORO) staining to detect the accumulation of neutral lipids in adipocytes at differentiation Day 8 (D8) (Figure 1C). The treatment of 2 µM compound **1** increased lipid accumulation by 1.8-fold compared with MDI-treated adipocytes, whereas compound **2**-treated adipocytes showed no significant difference from the control group. Compound **3** slightly increased the intracellular lipid content and compounds **4**, **5** and **6** did not show significant difference from control group. Rosiglitazone (1 µM) was used as control of a PPARγ agonist to increase adipogenesis.

### 2.2. Bavachin Regulates Proliferation and Differentiation of Adipocyte

**Figure 2:**
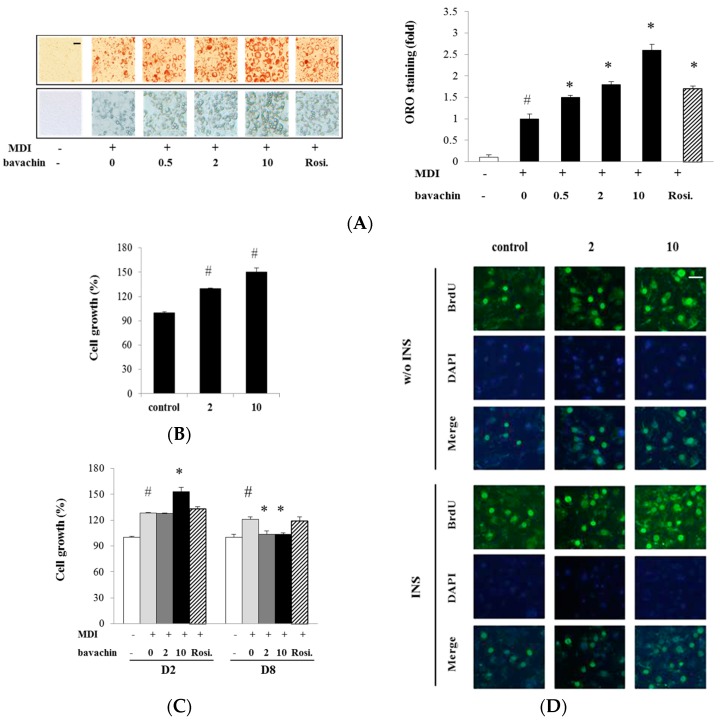
Effect of bavachin on proliferation and differentiation of adipocytes: (**A**) 3T3-L1 cells were differentiated with 0.5, 2 and 10 µM bavachin. Lipid accumulation was quantified by measuring absorbance of ORO staining. Images of adipocytes stained with ORO (**upper** panel, magnification, 40×) and differentiated adipocytes (**lower** panel, magnification, 100×) were visualized by light microscopy. Scale bar = 50 μm. Data are expressed as mean ± SD. ^#^ *p* < 0.001 *vs.* control; * *p* < 0.01 *vs.* MDI alone; (**B**) Effect of bavachin on the growth of pre-adipocyte. Pre-adipocytes were incubated with bavachin (2 and 10 µM) for 72 h, and cell viability was determined by MTT assay; (**C**) The pre-adipocytes incubated with bavachin (2 and 10 µM) in presence or absence of insulin (100 nM) were stained with BrdU. Immunofluorescence of BrdU-incorporated proliferating cells was visualized in green, and DAPI-labeled nuclei were seen as blue under light microscopy; (**D**) Cell growth of differentiated adipocytes was determined by MTT assay on differentiation Day 2 and 8 (D2 and D8). Scale bar = 200 μm. Data are expressed as mean ± SD. ^#^ *p* < 0.01 *vs.* control; * *p* < 0.05 *vs.* MDI.

As compound **1** (bavachin) showed both PPARγ transactivation and lipid accumulation activities, we further studied the adipogenic activity and mechanism of bavachin. As shown in Figure 2A, bavachin accumulated lipid in a dose dependent manner in ORO staining experiments. To determine whether bavachin affects cell growth or viability during differentiation, pre-adipocytes were subjected to the 3-(4,5-dimethylthiazol-2-yl)-2,5-diphenyltetrazolium bromide (MTT) and 5-bromo-2′-deoxy-uridine (BrdU) incorporation assays. Bavachin significantly increased the growth of preadipoctye at 10 µM compared with the control cells in MTT assay (Figure 2B). Bavachin also increased BrdU incorporation into newly synthesized DNA during pre-adipocyte proliferation (Figure 2C). BrdU incorporation was enhanced by insulin and further enhanced by co-treatment with insulin and bavachin at 2 and 10 µM.

Next, we investigated whether bavachin stimulates the growth of adipocytes during differentiation. As shown in Figure 2D, treatment of 10 µM bavachin significantly increased cell growth at D2, but significantly decreased growth of adipocytes at D8 compared with that of MDI-treated adipocytes.

In the present study, bavachin exhibited a different effect on growth and proliferation of pre-adipocytes and mature adipocytes.

### 2.3. Bavachin Activates Adipogenic Factors and Increases PPARγ Transcriptional Activity in Differentiated Adipocytes

**Figure 3:**
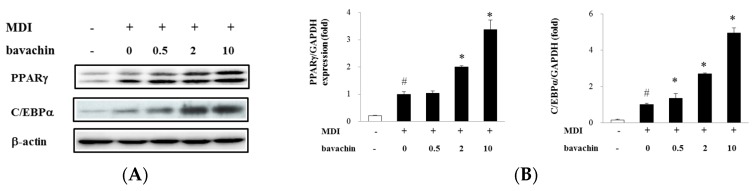

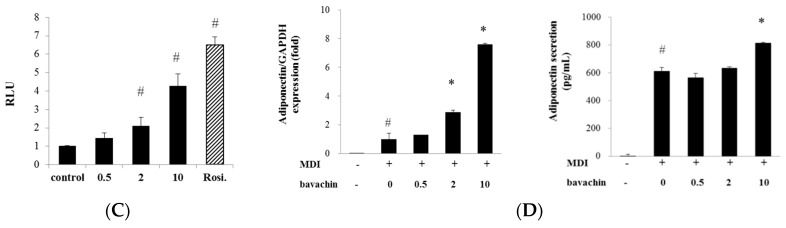
Effect of bavachin on the expression of adipogenic factors: (**A**) Effect of bavachin on PPARγ and C/EBPα protein levels of differentiated adipocytes. Pre-adipocytes were differentiated with or without bavachin at indicated concentrations. Cells were harvested on differentiation Day 5 (D5) and protein extracts (30 µg) were analyzed for PPARγ and C/EBPα expression by Western blot analysis. Actin was used as a loading control; (**B**) Effect of bavachin on the mRNA expression of PPARγ and C/EBPα. Differentiated adipocytes were harvested and lysed on D5. Gene expression levels of PPARγ and C/EBPα were determined by quantitative real-time polymerase chain reaction (qPCR); (**C**) Effect of bavachin on transactivation of PPARγ. PPARγ transactivation is presented as relative luciferase activity as described above; (**D**) Effect of bavachin on gene expression and secretion of adiponectin in differentiated adipocytes determined by real time RT-PCR and ELISA, respectively, as mentioned in the Materials and Methods. Data are expressed as mean ± SD. ^#^ *p* < 0.05 *vs.* control; * *p* < 0.05 *vs.* MDI.

We investigated the effect of bavachin on the adipogenic transcription factors, PPARγ and C/EBPα protein expression during adipocyte differentiation. As shown in Figure 3A, bavachin dose dependently increased PPARγ and C/EBPα expression. Quantitative real-time polymerase chain reaction (qPCR) experiments also showed that bavachin increased PPARγ and C/EBPα mRNA levels during differentiation in a dose dependent manner (Figure 3B).

We also evaluated the effect of bavachin on PPARγ-dependent transactivation. Bavachin dose dependently increased PPARγ-driven transcription showing 4.3-fold increment at 10 µM compared with control cells. Rosiglitazone was used as a positive control of PPRAγ agonist (Figure 3C). These data indicate that bavachin increased PPARγ expression, which in turn enhanced PPARγ-dependent transcription during adipocyte differentiation.

To confirm the insulin sensitizing effect of bavachin, we assessed the effect of bavachin on adiponectin excretion and mRNA expression during adipocyte differentiation (Figure 3D). Bavachin increased adiponectin mRNA level in a dose dependent manner. Secretion of adiponectin into culture media was also increased significantly by treatment with 10 µM bavachin, as compared with MDI treated cells.

### 2.4. Bavachin Enhances Insulin-Stimulated Glucose Uptake through GLUT4 Translocation via Akt and AMPK Pathway

**Figure 4:**
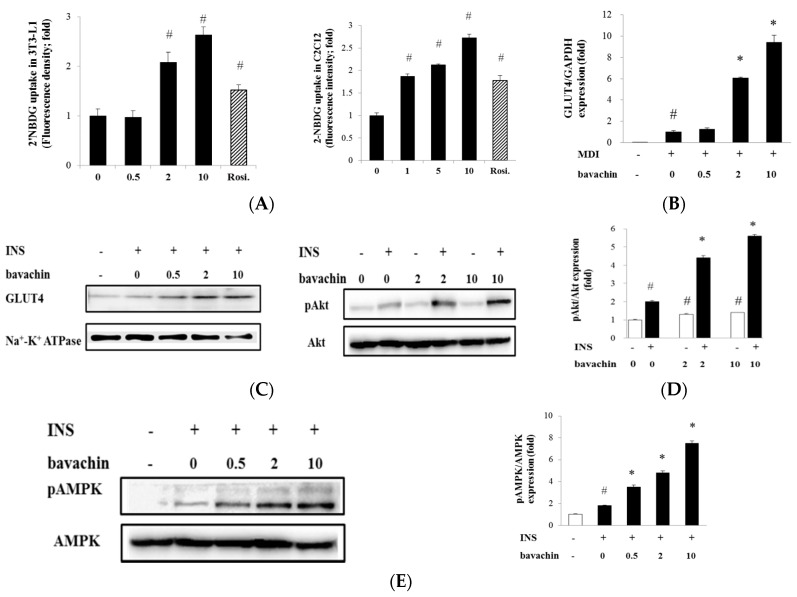
The effect of bavachin on insulin and AMPK signaling pathway-mediated glucose uptake: (**A**) the effect of bavachin on insulin-stimulated glucose uptake in differentiated adipocytes and myoblasts. Differentiated 3T3-L1 adipocytes (**left** panel) and C2C12 myoblasts (**right** panel) were serum-starved and then exposed to bavachin (0.5, 2 and 10 µM for adipocytes, 1, 5 and 10 nM for myoblasts) for 24 h. After 1 h incubation of insulin (100 nM), the cultures were incubated with 2-(*N*-(7-nitrobenz-2-oxa-1,3-diazol-4-yl) amino)-2-deoxyglucose (2-NBDG) labeling-medium for 1 h. Fluorescence retained in the cell monolayer was measured with excitation wavelength at 465 nm and emission wavelength at 540 nm. Data are expressed as mean ± SD. ^#^ *p* < 0.001 *vs.* insulin alone; (**B**) Effect of bavachin on the mRNA expression and translocation of GLUT4 in differentiated adipocyte. Gene expression level of GLUT4 was determined by qPCR as described above; (**C**) To detect the GLUT4 translocation, 3T3-L1 pre-adipocytes were pre-incubated for 1 h with bavachin and followed by insulin stimulation for a further 1 h. The plasma membrane fraction was prepared using an ultracentrifugation; The effect of bavachin on the phosphorylation of Akt (**D**) and AMPK (**E**) in adipocytes. 3T3-L1 pre-adipocytes were pre-treated for 1 h with bavachin prior to insulin (100 nM) for 1 h. Protein blots were incubated with phospho-Akt or phospho-AMPK antibody. Data are expressed as mean ± SD. ^#^ *p* < 0.01 *vs.* control; * *p* < 0.001 *vs.* insulin (INS).

We assessed the effects of bavachin on glucose uptake. As shown in Figure 4A, bavachin increased 2-(*N*-(7-nitrobenz-2-oxa-1,3-diazol-4-yl) amino)-2-deoxyglucose (2-NBDG) uptake by differentiated adipocytes and myoblasts in a dose-dependent manner. Rosiglitazone (1 µM) also significantly increased 2-NBDG uptake as a PPARγ agonist. Bavachin could improve insulin sensitivity via facilitating glucose uptake by differentiated adipocytes and myoblasts.

Next, we investigated the effect of bavachin on insulin-induced glucose transporter 4 (GLUT4) gene expression and translocation to plasma membrane. During adipocytes differentiation, bavachin dose dependently increased GLUT4 mRNA (Figure 4B) and protein expression (data not shown) compared with MDI-treated cells. To determine the effect of bavachin on GLUT4 translocation, we measured levels of GLUT4 in plasma membranes (Figure 4C). Insulin increased GLUT4 levels in the plasma membrane fraction and additional treatment with bavachin increased GLUT4 translocation up to 2.1-fold compared with the insulin-treated cells. These findings suggest that bavachin increased glucose uptake by activating expression and membrane translocation of GLUT4 in adipocytes.

To disclose the precise mechanism for glucose uptake, we assessed activation of the insulin signaling pathway by bavachin. Insulin increased Akt phosphorylation and bavachin dose dependently increased insulin-dependent Akt phosphorylation at 2 and 10 µM. Bavachin alone induced weak but significant increase in Akt phosphorylation, suggesting a direct effect of bavachin on Akt phosphorylation (Figure 4D).

As shown in Figure 4E, bavachin stimulated AMPK phosphorylation in insulin-stimulated adipocytes up to 4-fold at 10 µM compared with insulin-treated cells. These results indicate that bavachin augments insulin sensitivity via AMPK- and Akt-mediated pathways.

## 3. Discussion

Several reports have indicated that plant-derived anti-diabetic compounds induce intracellular lipid accumulation and enhance glucose uptake in 3T3-L1adipocytes [11]. Although anti-diabetic drugs, such as thiazolidinediones (TZD) improve insulin sensitivity [12], they are unfortunately associated with a risk of weight gain and hypoglycemia. Thus, new anti-diabetic agents with safe and minimal side effects are needed for anti-diabetic drug development.

PC extracts improve insulin sensitivity by free radical scavenging in the streptozotocin-induced diabetic animal model [5,6,13] and stimulate glucose uptake by yeast cells [7]. These beneficial effects of the PC extract resulted in an effort to identify potential anti-diabetic compounds. In our preliminary study, we observed that ethyl acetate fraction of PC stimulated adipogenesis in ORO staining experiments (Figure S1). Among the compounds isolated from ethyl acetate fraction, compounds **1**, **2** and **3** induced PPARγ transactivation in reporter gene assay. Bavachinin (compound **2**) was reported as a PPARγ ligand that directly interacted with ligand binding domain of PPARγ [14]. In ORO staining experiments performed as the functional assay of PPARγ ligand, bavachin (compound **1**) efficiently accumulated lipid, while bavachinin (compound **2**) did not show any significant difference from MDI control group. Since bavachin activated both PPARγ transcriptional activity and adipogenesis of adipocytes, we proposed bavachin as a promising insulin sensitizer in present study. Bavachin and bavachinin have same backbone structure that might be needed for PPARγ ligand activity, but only bavachin with hydroxy group at carbon number 7 activated the adipogenesis. The exact relationship between structure and adipogenic activity should be further studied.

Adipogenesis is mediated by the adipogenic transcriptional factors, PPARγ and C/EBPα [15,16]. Activation of PPARγ has been suggested to improve insulin sensitivity at a different step of insulin signaling pathway [17]. Therefore, PPARγ has been considered as a target for developing anti-diabetic drugs, such as TZD derivatives. PPARγ cooperates with C/EBPα to upregulate adipocyte-specific genes during adipocyte differentiation. Bavachin increased PPARγ and C/EBPα protein and mRNA expression (Figure 3A,B) and PPARγ transcriptional activity (Figure 3C), indicating that PPARγ might be involved in bavachin-stimulated adipogenesis.

Interestingly, bavachin increased proliferation of pre-adipocytes at the early stage of differentiation, but significantly decreased the growth of differentiated mature adipocytes (Figure 2). 3T3-L1 pre-adipocytes undergo cell division followed by growth arrest and expression of adipogenic genes just prior to differentiation. Therefore, regulating preadipoctye proliferation and cell cycle progression is a strategy for diabetes therapy. The TZD not only redistributes the abdominal fat mass, but also increases the number of small size adipocytes, which is associated with improved insulin sensitivity [18,19]. The neuropeptide orexin A improves insulin sensitivity by increasing proliferation of pre-adipocytes and subsequent reorganization of adipose tissue composition, not through stimulating differentiation [20]. Another study reported the stimulatory effects of orexina A on adipocyte differentiation [21]. These studies suggest different effects of orexin A on pre-adipocytes and mature adipocytes. Therefore, the effect of bavachin on adipocyte proliferation needs to be discussed further considering the contradictory effect of adipogenesis-stimulating agents on proliferation of pre-adipocytes and mature adipocytes.

Adiponectin is an adipokine secreted from adipocytes to regulate insulin sensitivity [22], and its low circulating levels are associated with insulin resistance and type 2 DM [23]. We found that bavachin stimulated adiponectin secretion from adipocytes during differentiation (Figure 3D)

TZD increases lipid storage in adipocytes and adipose tissues by increasing insulin-stimulated glucose uptake [24]. We found that bavachin increased the insulin-dependent uptake of 2-NBDG and GLUT4 mRNA level in differentiated adipocytes (Figure 4A). Among glucose transporters, GLUT4 is a major insulin-responsive transporter in adipocytes and the impaired translocation of GLUT4 increases the risk for DM [25]. Glucose uptake via GLUT4 could be controlled by factors including adiponectin or insulin signaling mediators [24]. Fu *et al.* [26] reported that adiponectin increases total expression and translocation of GLUT4 to plasma membrane in response to insulin. In this study, we demonstrated that bavachin induced translocation of GLUT4 to stimulate glucose uptake (Figure 4C).

We investigated whether bavachin could stimulate insulin-mediated GLUT4 translocation via Akt phosphorylation. Binding of insulin to its tyrosine kinase receptor induces the sequential phosphorylation of Akt and AS160, resulting in the accumulation of GTP-bound Rab proteins required for GLUT4 trafficking [27]. As expected, bavachin increased insulin-dependent Akt phosphorylation and showed marginally direct induction without insulin (Figure 4D). These data suggest that bavachin stimulates glucose uptake by activating the insulin-Akt signaling pathway.

Various extracts or chemicals from medicinal herbs improve glucose uptake via AMPK activation, which plays a central role in glucose and lipid metabolism in skeletal muscle and liver [28,29,30]. Metformin, an anti-diabetic drug, activates the AMPK pathway [31]. The cross-talk between PPARγ activation, insulin signaling, and AMPK activation modulates glucose uptake by recruiting GLUT4 to the plasma membrane. Bavachin from PC significantly activated the insulin signaling pathway and AMPK, leading to stimulation of glucose uptake by adipocytes. In summary, bavachin purified from *Psoralea corylifolia* induced adipocyte differentiation and enhanced glucose uptake via GLUT4 translocation to plasma membrane through activating the Akt and AMPK pathway. Bavachin might have therapeutic potential for type 2 diabetes by activating insulin signaling pathways.

## 4. Materials and Methods

### 4.1. Isolation of Compound from Psoralea corylifolia L. (PC)

The dried seeds of PC were purchased from the Kyungdong Oriental Drug Market in 2013 (Seoul, Korea). A voucher specimen (No. SPH 13003) was deposited in the herbarium of Sookmyung Women’s University (Seoul, Korea). The air-dried plant materials (8.8 kg) were extracted with n-hexane (1.1 kg), and the remaining material was extracted with methanol. After the extracted solution was filtered and evaporated *in vacuo*, the residue (1.6 kg) was suspended in water and successively partitioned with hexane and ethyl acetate. The ethyl acetate fraction (153 g) was subjected to silica and RP-C18 (LiChroprep RP-C18, 40–63 µm, Merck, Darmastadt, Germany) column chromatography and six compounds were isolated: bavachin (**1**) [32], bavachinin (**2**) [32], 7,8-dihydro-8-(4-hydrxyophenyl)-2,2-dimethyl-2*H*,6*H*-benzo-[1,2-b:5,4-b′]dipyran-6-one (**3**) [32], corylin (**4**) [33], kanzonol B (**5**) [34], and bakuchiol (**6**) [33]. Purity of all compounds was confirmed by reverse-phase high performance liquid chromatography analysis and the nuclear magnetic resonance (NMR) spectrum. Structures were elucidated by IR, mass, and NMR spectroscopic data analysis.

### 4.2. Cell Culture and Pre-Adipocyte Differentiation

Mouse 3T3-L1 pre-adipocytes (American Type Culture Collection, Manasas, VA, USA) and C2C12 myoblasts (kindly provided by Gyu-Un Bae, Sookmyung Women’s University, Seoul, Korea) were maintained and differentiated according to the previously described methods [11,35]. Two days after pre-adipocytes reached confluence (differentiation Day 0, D0), the medium was replaced with media containing MDI (1 µg/mL isobutyl-methylxanthine, 1 µM dexamethasone, and 1 µg/mL insulin (Sigma, St. Louis, MO, USA)). After two days incubation (D2), the MDI medium was replaced with insulin-containing DMEM. During differentiation, cells were maintained by replenishing with new MDI medium every two days. To induce differentiation of C2C12 myoblasts, cells at near confluence were cultured with DMEM containing 2% horse serum [35] until myotube formation was observed (normally at 2–3 days of differentiation).

### 4.3. Oil Red O (ORO) Staining and Microscopy of Lipid Drop Formation in 3T3-L1 Cells

The lipid content of differentiated 3T3-L1 cells was evaluated using ORO staining method at D8. Absorbance of the extract was measured at 520 nm using a GloMax^®^-Multi Microplate Multimode Reader (Promega, Madison, WI, USA). Lipid drop accumulation in 3T3-L1 cells was photographed using an inverted phase-contrast microscope (TH4, Olympus, Tokyo, Japan).

### 4.4. 3-(4,5-Dimethylthiazol-2-yl)-2,5-diphenyltetrazoliumbromide (MTT) and Cell Proliferation Assay

To determine the effect of bavachin on pre-adipocyte viability, 3T3-L1 pre-adipocytes were incubated with different concentration of bavachin for 72 h. The cells were subjected to MTT assay (Sigma). Cell proliferation assays were performed using the 5-bromo-2′-deoxy-uridine (BrdU) Labeling and Detection Kit I (Roche, Mannheim, Germany), according to the manufacturer’s instructions. Briefly, pre-adipocytes were incubated in serum-free medium for 12 h and then treated with bavachin in the presence or absence of insulin for another 24 h. Cells were switched to BrdU-labeling medium and incubated for an additional 3 h. Bound anti-BrdU-Fluorescein was detected by Olympus fluorescence microscopy.

### 4.5. Glucose Uptake Assay

The glucose uptake assay was performed according to the manufacturer’s instructions with minor modifications. After differentiation of 3T3-L1 pre-adipocytes and C2C12 myoblasts, the cells were treated with bavachin for 24 h. After 1 h incubation of insulin, 20 µM of the fluorescent glucose analog 2-(*N*-(7-nitrobenz-2-oxa-1,3-diazol-4-yl) amino)-2-deoxyglucose (2-NBDG) was added, and the fluorescence retained in the cell monolayer was measured using a microplate reader with excitation wavelength at 465 nm and emission wavelength at 540 nm.

### 4.6. Adiponectin Secretion Assay

At D5, following differentiation, the conditioned medium was collected and adiponectin concentration in conditioned medium was measured using the Mouse Adiponectin/Acrp30 DuoSet (R&D Systems, Minneapolis, MN, USA).

### 4.7. Peroxisome Proliferator-Activated Receptor (PPAR)γ Reporter Gene Assay

CV-1 cells were transiently transfected with a plasmid mixture containing a PPARγ expression vector and the tk-PPRE-luciferase (Luc) vector, and then treated with test sample for 24 h. Luciferase activity in cell lysates was measured using the luciferase assay system (Promega). Data are reported as relative luciferase activity divided by β-galactosidase activity. All constructs were kindly gifted by Ronald M. Evans (The Salk Institute, La Jolla, CA, USA).

### 4.8. RNA Extraction and Quantitative Real-Time Reverse Transcription Polymerase Chain Reaction (qPCR)

To estimate gene expression levels during adipocyte differentiation, qPCR reactions were performed with the SYBR^®^ Green PCR Master Mix and conducted using the Applied Biosystems 7500 Fast Real-Time PCR System (Foster City, CA, USA). All mRNA levels were normalized using glyceraldehyde 3-phosphate dehydrogenase mRNA as an internal control. The primers used for amplifications are shown in Table 1.

### 4.9. Preparation of the Plasma Membrane Fraction and Western Blot Analysis

To determine the effect of bavachin on protein expression of PPARγ, C/EBPα and GLUT4, cells were lysed at D5. To detect Akt and AMPK activation and GLUT4 translocation, 3T3-L1 pre-adipocytes were pre-incubated for 1 h with bavachin followed by insulin stimulation for another 1 h. Total protein was electrophoresed in SDS-polyacrylamide gels and transferred to polyvinylfluoride membranes. The membrane was probed with primary antibodies against PPARγ, C/EBPα, phospho-Akt, phospho-AMPK, or GLUT4 (Cell Signaling Technology, Danvers, MA, USA). The plasma membrane fraction was prepared using a modified ultracentrifugation method [35].

### 4.10. Statistical Analysis

Data are expressed as mean ± standard deviation and differences were assessed using Student’s *t*-test. All experiments were conducted at least three times. A *p*-value <0.05 was considered significant.

## 5. Conclusions

Bavachin purified from PC enhanced glucose uptake via GLUT4 translocation by activating the Akt and AMPK pathway in the presence or absence of insulin. These results suggest that bavachin might have therapeutic potential for type 2 diabetes by activating insulin signaling pathways and AMPK.

## Figures and Tables

**Table 1:** Oligonucleotide primer sequences used for the qRT-PCR analysis.

Gene Name	Forward Primer (5–3)	Reverse Primer (5–3)	Accession Number
PPARγ2	AACTCTGGGAGATTCTCCTGTTGA	GAAGTGCTCATAGGCAGTGCAT	EF062476
C/EBPα	TGCTGGAGTTGACCAGTAC	AAACCATCCTCTGGGTCTCC	NM_001287523
Adiponectin	TGTAGGATTGTCAGTGGATCTG	GCTCTTCAGTTGTAGTAACGTCATC	AY749429
GLUT4	GGGTCCTTACGTCTTCCTTCT	CCTCTGGTTTCAGGCACTTT	NM_009204
GAPDH	TGCACCACCAACTGCTTAG	GGCATGGACTGTGGTCATGAG	BC096042

PPARγ2, peroxisome proliferator activated receptor subtype γ 2; *C/EBPα*, CCAAT/enhancer binding protein-α; GLUT4, glucose transporter subtype 4; GAPDH, glyceraldehyde 3-phosphate dehydrogenase.

## Supplementary Materials

Supplementary materials can be found at http://www.mdpi.com/1422-0067/17/4/527/s1.

## References

[1] Kwon D.Y., Kim da S., Yang H.J., Park S. (2011). The lignan-rich fractions of Fructus Schisandrae improve insulin sensitivity via the PPAR-γ pathways in *in vitro* and *in vivo* studies. J. Ethnopharmacol..

[2] Joo J.I., Kim D.H., Yun J.W. (2010). Extract of Chaga mushroom (*Inonotus obliquus*) stimulates 3T3-L1 adipocyte differentiation. Phytother. Res..

[3] Khushboo P.S., Jadhav V.M., Kadam V.J., Sathe N.S. (2010). *Psoralea corylifolia* Linn.—“Kushtanashini”. Pharmacogn. Rev..

[4] Kamboj J., Sharma S., Kumar S. (2011). *In vivo* Anti-diabetic and anti-oxidant potential of *Psoralea corylifolia* seeds in Streptozotocin induced type-2 diabetic rats. J. Health Sci..

[5] Seo E., Lee E.K., Lee C.S., Chun K.H., Lee M.Y., Jun H.S. (2014). *Psoralea corylifolia* L. seed extract ameliorates streptozotocin-induced diabetes in mice by inhibition of oxidative stress. Oxid. Med. Cell. Longev..

[6] Bera T.K., Ali K.M., Jana K., Ghosh A., Ghosh D. (2013). Protective effect of aqueous extract of seed of *Psoralea corylifolia* (Somraji) and seed of *Trigonella foenum-graecum* L. (Methi) in streptozotocin-induced diabetic rat: A comparative evaluation. Pharmacogn. Res..

[7] Suhashini R., Sindhu S., Sagadevan E. (2014). *In vitro* evaluation of anti diabetic potential and phytochemical profile of *Psoralea corylifolia* seeds. Int. J. Pharmacogn. Phytochem. Res..

[8] Krenisky J.M., Luo J., Reed M.J., Carney J.R. (1999). Isolation and antihyperglycemic activity of bakuchiol from *Otholobium pubescens* (Fabaceae), a Peruvian medicinal plant used for the treatment of diabetes. Biol. Pharm. Bull..

[9] Cecilia L., Faini F., Joseph C., Hoshph D.C. (1996). Bakuchiol derivatives from the leaves of *Psoalea grandulosa*. Phytochemistry.

[10] Wang D., Li F., Jiang Z. (2001). Osteoblastic proliferation stimulating activity of *Psoralea corylifolia* extracts and two of its flavonoids. Planta Med..

[11] Vaidya H., Goyal R.K., Cheema S.K. (2013). Anti-diabetic activity of swertiamarin is due to an active metabolite, gentianine, that upregulates PPAR-γ gene expression in 3T3-L1 cells. Phytother. Res..

[12] Yamauchi T., Kamon J., Waki H., Murakami K., Motojima K., Komeda K., Ide T., Kubota N., Terauchi Y., Tobe K. (2001). The mechanisms by which both heterozygous peroxisome proliferator-activated receptor γ (PPARγ) deficiency and PPARγ agonist improve insulin resistance. J. Biol. Chem..

[13] Chopra B., Dhingra A.K., Dhar K.L. (2013). *Psoralea corylifolia* L. (Buguchi)—Folklore to modern evidence: Review. Fitoterapia.

[14] Du G., Feng L., Yang Z., Shi J., Huang C., Guo F., Li B., Zhu W., Li Y. (2015). Separation and peroxisome proliferator-activated receptor-γ agonist activity evaluation of synthetic racemic bavachinin enantiomers. Bioorg. Med. Chem. Lett..

[15] Cho K.W., Lee O.H., Banz W.J., Moustaid-Moussa N., Shay N.F., Kim Y.C. (2010). Daidzein and the daidzein metabolite, equol, enhance adipocyte differentiation and PPARγ transcriptional activity. J. Nutr. Biochem..

[16] Tang Q.Q., Otto T.C., Lane M.D. (2003). CCAAT/enhancer-binding protein β is required for mitotic clonal expansion during adipogenesis. Proc. Natl. Acad. Sci. USA.

[17] Leonardini A., Laviola L., Perrini S., Natalicchio A., Giorgino F. (2009). Cross-talk between PPARγ and insulin signaling and modulation of insulin sensitivity. PPAR Res..

[18] De Souza C.J., Eckhardt M., Gagen K., Dong M., Chen W., Laurent D., Burkey B.F. (2001). Effects of pioglitazone on adipose tissue remodeling within the setting of obesity and insulin resistance. Diabetes.

[19] Takamura T., Nohara E., Nagai Y., Kobayashi K. (2001). Stage-specific effects of a thiazolidinedione on proliferation, differentiation and PPARγ mRNA expression in 3T3-L1 adipocytes. Eur. J. Pharmacol..

[20] Skrzypski M., Kaczmarek P., Le T.T., Wojciechowicz T., Pruszynska-Oszmalek E., Szczepankiewicz D., Sassek M., Arafat A., Wiedenmann B., Nowak K.W. (2012). Effects of orexin A on proliferation, survival, apoptosis and differentiation of 3T3-L1 preadipocytes into mature adipocytes. FEBS Lett..

[21] Skrzypski M., Le T.T., Kaczmarek P., Pruszynska-Oszmalek E., Pietrzak P., Szczepankiewicz D., Kolodziejski P.A., Sassek M., Arafat A., Wiedenmann B. (2011). Orexin A stimulates glucose uptake, lipid accumulation and adiponectin secretion from 3T3-L1 adipocytes and isolated primary rat adipocytes. Diabetologia.

[22] Kopp C., Hosseini A., Singh S.P., Regenhard P., Khalilvandi-Behroozyar H., Sauerwein H., Mielenz M. (2014). Nicotinic acid increases adiponectin secretion from differentiated bovine preadipocytes through G-protein coupled receptor signaling. Int. J. Mol. Sci..

[23] Whitehead J.P., Richards A.A., Hickman I.J., Macdonald G.A., Prins J.B. (2006). Adiponectin—A key adipokine in the metabolic syndrome. Diabetes Obes. Metab..

[24] Litherland G.J., Hajduch E., Hundal H.S. (2001). Intracellular signalling mechanisms regulating glucose transport in insulin-sensitive tissues (review). Mol. Membr. Biol..

[25] Sano H., Eguez L., Teruel M.N., Fukuda M., Chuang T.D., Chavez J.A., Lienhard G.E., McGraw T.E. (2007). Rab10, a target of the AS160 Rab GAP, is required for insulin-stimulated translocation of GLUT4 to the adipocyte plasma membrane. Cell Metab..

[26] Fu Y., Luo N., Klein R.L., Garvey W.T. (2005). Adiponectin promotes adipocyte differentiation, insulin sensitivity, and lipid accumulation. J. Lipid Res..

[27] Eguez L., Lee A., Chavez J.A., Miinea C.P., Kane S., Lienhard G.E., McGraw T.E. (2005). Full intracellular retention of GLUT4 requires AS160 Rab GTPase activating protein. Cell Metab..

[28] Ha do T., Trung T.N., Hien T.T., Dao T.T., Yim N., Ngoc T.M., Oh W.K., Bae K. (2010). Selected compounds derived from Moutan Cortex stimulated glucose uptake and glycogen synthesis via AMPK activation in human HepG2 cells. J. Ethnopharmacol..

[29] Lee M.S., Hwang J.T., Kim S.H., Yoon S., Kim M.S., Yang H.J., Kwon D.Y. (2010). Ginsenoside Rc, an active component of *Panax ginseng*, stimulates glucose uptake in C2C12 myotubes through an AMPK-dependent mechanism. J. Ethnopharmacol..

[30] Russo G.L., Russo M., Ungaro P. (2013). AMP-activated protein kinase: A target for old drugs against diabetes and cancer. Biochem. Pharmacol..

[31] Gasparrini M., Giampieri F., Alvarez Suarez J.M., Mazzoni L., Forbes Hernandez T.Y., Quiles J.L., Bullon P., Battino M. (2015). AMPK as a new attractive therapeutic target for disease prevention: The role of dietary compounds. Curr. Drug Targets.

[32] Lee M.H., Kim J.Y., Ryu J.H. (2005). Prenylflavones from *Psoralea corylifolia* inhibit nitric oxide synthase expression through the inhibition of I-κB-α degradation in activated microglial cells. Biol. Pharm. Bull..

[33] Lee S.W., Yun B.R., Kim M.H., Park C.S., Lee W.S., Oh H.M., Rho M.C. (2012). Phenolic compounds isolated from *Psoralea corylifolia* inhibit IL-6-induced STAT3 activation. Planta Med..

[34] Ngadjui B.T., Watchueng J., Keumedjio F., Ngameni B., Simo I.K., Abegaz B.M. (2005). Prenylated chalcones, flavone and other constituents of the twigs of *Dorstenia angusticornis* and *Dorstenia barteri* var. subtriangularis. Phytochemistry.

[35] Hwang J., Lee S.J., Yoo M., Go G.Y., Lee da Y., Kim Y.K., Seo D.W., Kang J.S., Ryu J.H., Bae G.U. (2015). Kazinol-P from Broussonetia kazinoki enhances skeletal muscle differentiation via p38MAPK and MyoD. Biochem. Biophys. Res. Commun..

