# Supplementary material for: Bavachin from Psoralea corylifolia Improves Insulin-Dependent Glucose Uptake through Insulin Signaling and AMPK Activation in 3T3-L1 Adipocytes
Source: Int J Mol Sci. 2016 Apr 8;17(4):527. doi: 10.3390/ijms17040527 (PMC4848983; doi:10.3390/ijms17040527)
Supplement: Supplementary file 1 [file ijms-17-00527-s001.pdf]

Supplementary Materials: Bavachin from *Psoralea corylifolia* Improves Insulin-Dependent Glucose Uptake through Insulin Signaling and AMPK Activation in 3T3-L1 Adipocytes

Hyejin Lee, Hua Li, Minsoo Noh and Jae-Ha Ryu

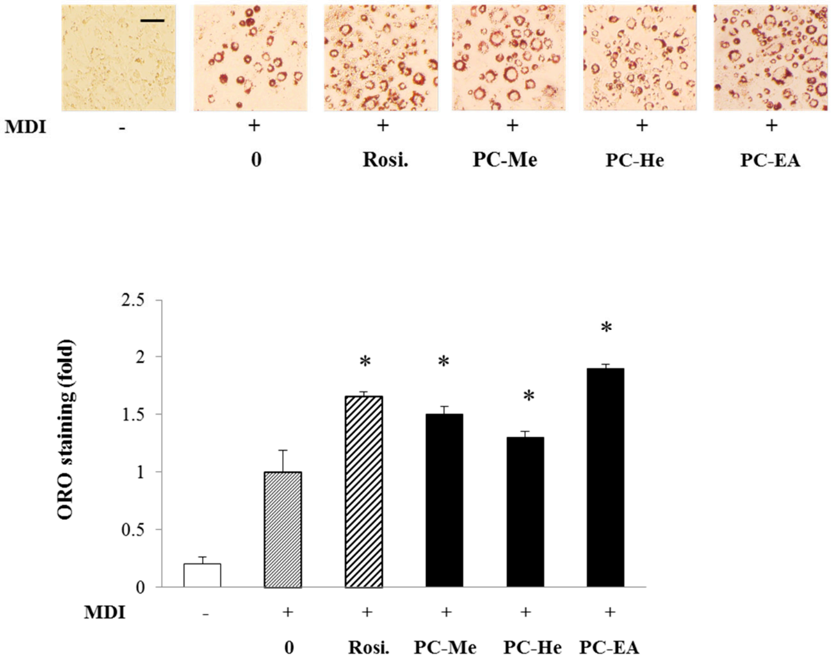

Figure S1. Effect of extract and fractions of *Psoralea corylifolia* (PC) on adipocyte differentiation. 3T3-L1 cells were differentiated in the presence of test materials. At differentiation Day 8 (D8), cells were stained with Oil Red-O (ORO), and lipid accumulation was quantified as described in Materials and Methods section. Scale bar = 50 μm. Data are expressed as mean ± standard deviation (SD). 0, MDI alone; Rosi., rosiglitazone (1 μM); PC-Me, methanol extract; PC-He, hexane fraction; PC-EA, ethyl acetate fraction (0.5 μg/mL each). * $p < 0.01$ *vs.* MDI.
